# The first geophilid centipedes from Malesia: a new genus with two new species from Sumatra (Chilopoda, Geophilidae)

**DOI:** 10.3897/zookeys.605.9338

**Published:** 2016-07-14

**Authors:** Lucio Bonato, Bernhard Klarner, Rahayu Widyastuti, Stefan Scheu

**Affiliations:** 1 Università di Padova, Dipartimento di Biologia, Via Bassi 58B, I-35131 Padova, Italy; 2 Georg August University Göttingen, J.F. Blumenbach Institute of Zoology and Anthropology, Berliner Str. 28, 37073 Göttingen, Germany; 3 Institut Pertanian Bogor - IPB, Department of Soil Sciences and Land Resources, Damarga Campus, Bogor 16680, Indonesia

**Keywords:** Chilopoda, forcipules, Geophilidae, Southeast Asia, Sundageophilus

## Abstract

A new genus *Sundageophilus* is here described for two new species of geophilid centipedes (Chilopoda: Geophilidae) from Sumatra, Indonesia. Both *Sundageophilus bidentatus* **sp. n.** and *Sundageophilus poriger* **sp. n.** feature a minute body size (less than 1 cm long with 31–35 pairs of legs), a similar structure of the maxillae, elongated forcipules, and few coxal organs. *Sundageophilus bidentatus* is unique among geophilids because the ultimate article of the forcipule is armed with two conspicuous denticles, one dorsal to the other, instead of a single one or none. The two species of *Sundageophilus* are the first genuine Geophilidae ever found in Malesia, and among the very few representatives of this family in the entire south-eastern Asia.

## Introduction

The diversity of geophilomorph centipedes (Geophilomorpha) in south-eastern Asia is still largely unexplored. In comparison with other areas, including nearby tropical regions, these soil arthropods have remained notably under-sampled in the entire Indochina, Malesia, and Papuasia. Many naturalistic expeditions reached these lands and islands between the late 19^th^ and early 20^th^ centuries, but they provided only few specimens and records (Attems 1914). Some advances were subsequently contributed by various taxonomists (Chamberlin 1920, 1939, 1944; Attems 1930a, 1938, 1953; Verhoeff 1937; Würmli 1972; Lewis 1991); however, to date all records from this broad area are based on a small number of samples from a few sparse localities. Additionally, records are biased towards epigeic and larger-bodied species. For a recent overview see Bonato and Zapparoli (2011).

The situation is especially unsatisfactory for the large island of Sumatra, when compared with the neighbouring Malay peninsula and the other Malesian islands. To the best of our knowledge, all records of geophilomorph centipedes from Sumatra derive from half a dozen papers (Pocock 1894; Silvestri 1895, 1919; Attems 1930b; Chamberlin 1944; Lewis 1991) and concern specimens collected in no more than a dozen localities. Most of the species hitherto recorded belong to Mecistocephalidae, Oryidae and Gonibregmatidae, which are relatively large and conspicuous geophilomorphs, the only small sized exception is a species of Ballophilinae (Table 1). No concrete evidence has been obtained to date for Sumatra and even for Malesia as a whole, for the presence of species of Geophilidae, which is by far the richest and most widespread family of geophilomorph centipedes in the world (Bonato and Zapparoli 2011).

**Table 1.** Species of Geophilomorpha hitherto recorded from Sumatra and published sources of records. For the taxonomic names and classification, we referred to Bonato et al. (2016). Published taxonomic names different from the current ones are given in squared parentheses.

Species	Source/s
Mecistocephalidae	
*Tygarrup* sp. (at least one species, possibly more than one)	Pocock 1894 [*Mecistocephalus spissus*] Silvestri 1895 [*Mecistocephalus spissus*] possibly Attems 1930a [*Mecistocephalus spissus*]
*Mecistocephalus* sp. (at least two species)	Haase 1887 [*Mecistocephalus punctifrons*] Pocock 1894 [*Mecistocephalus punctifrons*] Silvestri 1895 [*Mecistocephalus punctifrons*] Attems 1914 [*Mecistocephalus insularis*] Silvestri 1919 [*Lamnonyx insularis* varietas *orientalis*; *Lamnonyx cephalotes* varietas *subinsularis*] Verhoeff 1937 [*Mecistocephalus verrucosus*] Lewis 1991 [*Mecistocephalus verrucosus*]
Oryidae	
*Orphnaeus brevilabiatus* (Newport,1845)	Silvestri 1895 Attems 1930a Chamberlin 1944
Schendylidae Ballophilinae	
*Ballophilus pedadanus* Chamberlin, 1944	Chamberlin 1944 Lewis 1991
Gonibregmatidae	
*Geoporophilus angustus* Silvestri, 1919	Silvestri 1919
*Geoporophilus aporus* Attems, 1930	Attems 1930a
*Himantosoma porosum* Pocock, 1891	Silvestri 1895 [later described as *Himantosoma typicum* varietas *tridivisum*; Silvestri 1919]

Two new species of Geophilomorpha are described from Sumatra. They are representatives of a new lineage of minute animals that have hitherto escaped the attention of myriapodologists and have evolved a previously unknown pattern of forcipular denticles. They are the first Geophilidae ever found in Malesia, and among the very few representatives of this family recorded in the entire south-eastern Asia, from Indochina to Papuasia.

## Material and methods

Specimens were found in soil samples collected in Sumatra, along a gradient including secondary rainforests, jungle rubber agroforests, rubber, and oil palm plantations. Sampling has been carried out within the interdisciplinary project “Ecological and socioeconomic functions of tropical lowland rainforest transformation systems (Sumatra, Indonesia) – EFForTS”. For details on the study region and experimental design see Drescher et al. (2016). Specimens were extracted from soil cores by heat (Kempson et al. 1963) and fixed in 70% ethanol.

The specimens were examined by light microscopy (Leica DMLB) according to standard protocols for geophilomorphs, by placing them in ethylene glycol in temporary mounts (Pereira 2000). The head was detached from the trunk for some specimens only. Measurements were taken using a micrometre eyepiece. Digital photographs were taken using a camera (Leica DFC420) attached to the microscope and assembled using an image stacking software (CombineZP; Hadley 2008). Line-drawings were produced manually from the photographs. For the morphological terminology, we followed Bonato et al. (2010).

To evaluate whether similar or possibly related species were already recorded in south-eastern Asia, the entire taxonomic and faunistic literature on centipedes was browsed to retrieve all published records from that area.

### Abbreviations

LIPI
 Indonesian Institute of Science, Cibinong, Indonesia 


PD
 Department of Biology, University of Padova, Italy 


## Taxonomy

### Sundageophilus gen. n.

Taxon classificationAnimaliaGeophilomorphaGeophilidae

http://zoobank.org/11CB8320-AC3E-4657-B217-AB6E30459855

#### Diagnosis.

Relatively small geophilids, less than 1 cm long; cephalic plate distinctly elongate, without frontal line; clypeus with two pairs of setae on the anterior medial part, without a distinct clypeal area; intermediate part of labrum bearing stout tubercles, lateral parts far apart from each other and bearing bristles; first maxillae without lappets; second maxillary coxosternite with anterior margin entire and concave, without anterior projections, neither statuminia nor other distinctly sclerotized parts associated with the metameric pores; second maxillary pretarsus in shape of an elongate claw, more than 3.5 times as long as wide at the basis, sub-conic and slightly bent, with a small sub-basal dorsal bulge; forcipular tergite subtrapezoidal; forcipular coxosternite relatively elongate, the exposed part as wide as or only slightly wider than long, the anterior margin slightly projecting anteriorly, with two very short denticles and a narrow notch inbetween; coxopleural sutures complete, entirely ventral, straight and subparallel for most of their length; chitin-lines absent or hardly distinct; forcipules relatively elongate, the trochanteroprefemur is more than 1.4 times as long as wide, the tarsungulum more than 2.5 times as long as wide; forcipular intermediate articles distinct, without denticles; tarsungulum with at least a distinct basal denticle; trunk metasternites longer than wide, without obvious “carpophagus” pit; whenever present, a single sub-circular, posterior pore-field on all metasternites of the trunk; leg claws with at most a pair of accessory spines, shorter than mid-length of the pretarsus, similar to each other in length; ultimate leg-bearing segment with an entire pleuropretergite, without sulci; ultimate metasternite sub-trapezoid, the setae distributed almost uniformly in the female, unknown in the male; coxopleuron with at least two coxal organs, opening through independent pores on the ventral side; telopodite of the ultimate leg pair approximately 1.8–2.0 times as long as that of the penultimate pair; anal pores distinct.

#### Etymology.

From “Sunda”, the name in use for the south-eastern part of the continental shelf of Asia, including Sumatra and other islands, and “Geophilus”, which is used in many other names of genera of geophilids.

#### Type species.

*Sundageophilus bidentatus* sp. n.

### Sundageophilus bidentatus sp. n.

Taxon classificationAnimaliaGeophilomorphaGeophilidae

http://zoobank.org/96F28A97-E612-4D89-A8CC-99A8F270A5BC

#### Diagnosis.

A *Sundageophilus* species with cephalic plate ca. 1.4–1.5 times as long as wide; first maxillary telopodite apparently composed of a single article; a distinct denticle on the distal part of the trochanteroprefemur; tarsungulum with two basal denticles, one dorsal to the other; 33 or 35 pairs of legs; no ventral pore-fields along the trunk; ultimate metasternite 1.7–1.8 times as wide as long, anteriorly ca. 2.0 times as wide as posteriorly, lateral margins slightly convex; two coxal pores on each coxopleuron, along the lateral margin of the metasternite; telopodites of the ultimate pair conspicuously swollen in the female, unknown in the male, apparently composed of only five articles because of the indistinct articulation between trochanter and prefemur; some articles of the ultimate leg pair with disto-ventral bulges covered with denser, longer setae, and a tuft of tiny spines replacing the pretarsus.

#### Material examined.

*Holotype*. ♀ with gonopods partially developed, body length 6.9 mm, head width 220 µm; some legs broken and missing, including one of the ultimate pair; originally entire, subsequently divided in three pieces, (i) head, (ii) forcipular segment and leg-bearing segments 1–16, (iii) leg-bearing segments 17–33 and postpedal segments; collected Nov. 2013, by B. Klarner; in ethanol, LIPI macrosoilBO4a13_chilo144.

*Type locality*. Indonesia, Sumatra, Bukit Duabelas, oil palm plantation, research site BO4, 02°03'02"S, 102°45'12"E, ca. 30 m a.s.l., from upper soil layer (0–5 cm).

*Other specimens examined*. 1 ♀, from Bukit Duabelas, secondary rainforest, research site BF2, 01°58'55"S, 102°45'03"E, ca. 80 m a.s.l., from upper soil layer (0–5 cm), same date and collector as holotype, PD5768; 6 ♀♀, from Bukit Duabelas, secondary rainforest, research site BF3, 01°56'34"S, 102°34'53"E, ca. 90 m a.s.l., from upper soil layer (0–5 cm), same date and collector as holotype, LIPI macrosoilBF3a13_chilo178–183; 1 ♀, from Harapan, secondary rainforest, research site HF3, 02°10'30"S, 103°19'58"E, ca. 60 m a.s.l., from upper soil layer (0–5 cm), same date and collector as holotype, LIPI macrosoilHF3c13_chilo17; 1 specimen, sex unknown because body posterior part missing, from Harapan, jungle rubber agroforest, research site HJ2, 01°49'32"S, 103°17'39"E, ca. 80 m a.s.l., from upper soil layer (0–5 cm), same date and collector as holotype, PD5767.

#### Etymology.

“*bidentatus*” means “with two teeth” and refers to the presence of two distinct basal denticles on each forcipular tarsungulum.

#### Description.

**Figure 1.**
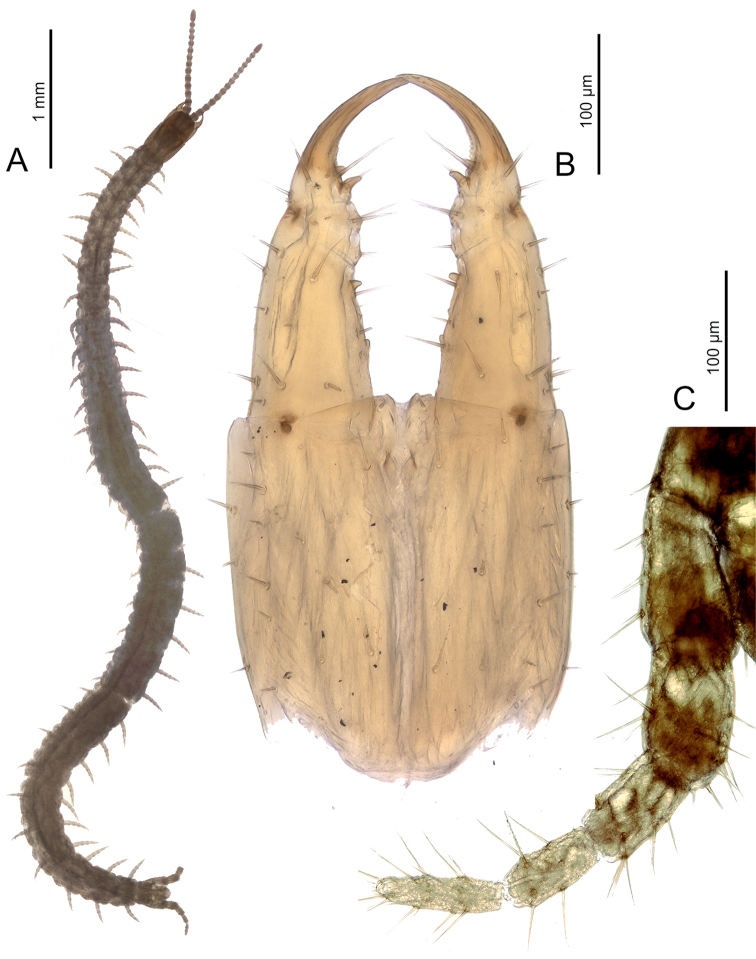
*Sundageophilus bidentatus* sp. n.: **A** entire body, dorsal view **B** forcipular segment, ventral view **C** ultimate left leg, dorsal view. LM photos of holotype LIPI macrosoilBO4a13_chilo144 (**A, C**) and PD5768 (**B**).

**Figure 2.**
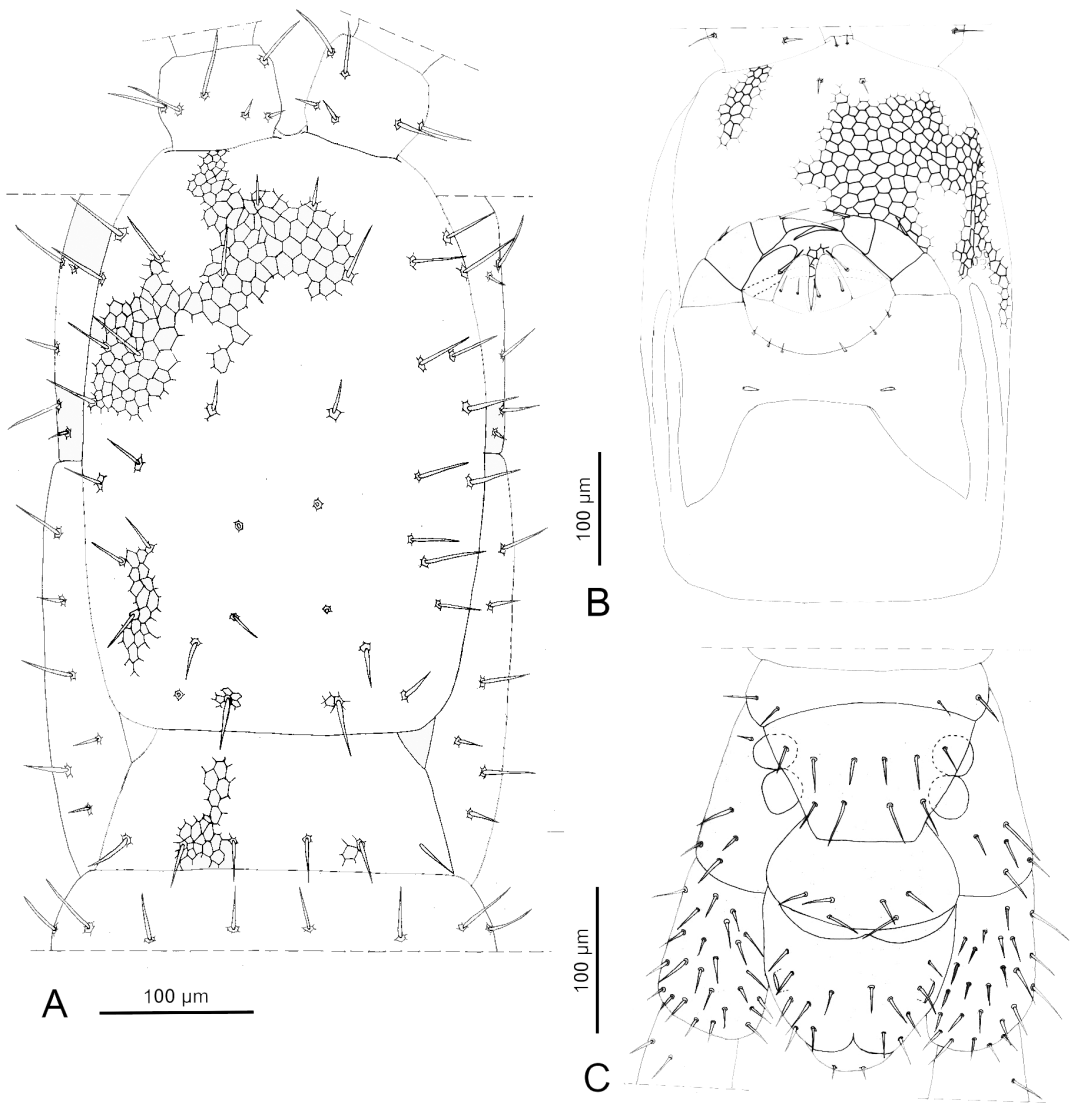
*Sundageophilus bidentatus* sp. n.: **A** head and forcipular segment, dorsal view, antennal articles II–XIV and tips of forcipules omitted, areolation only partially drawn **B** head, ventral view, areolation only partially drawn **C** ultimate leg-bearing segment and postpedal segments, ventral view, ultimate legs partially omitted. Line drawings based on LM photos of holotype LIPI macrosoilBO4a13_chilo144.

**Figure 3.**
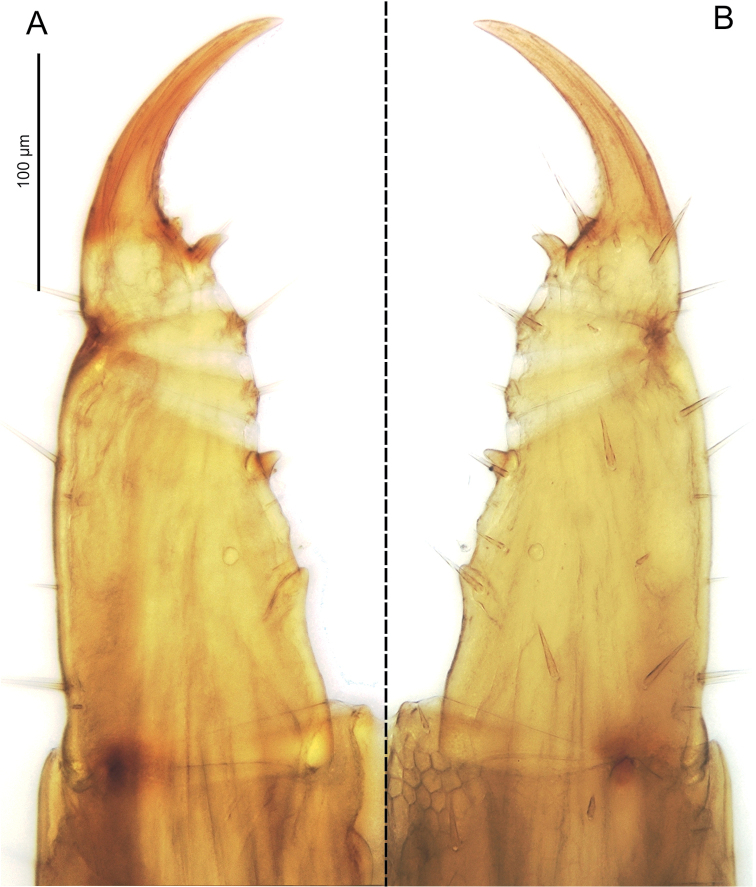
*Sundageophilus bidentatus* sp. n.: left forcipule, dorsal (**A**) and ventral (**B**) views. LM photos of holotype LIPI macrosoilBO4a13_chilo144.

*Description of holotype* (♀, LIPI macrosoilBO4a13_chilo144). See also Figs 1A, 1C, 2, 3.

*General features*. Body remarkably narrow, almost uniformly wide for most part of the trunk, only slightly narrowing posteriorly. Legs relatively short, most of them ca. 0.2 mm long. Colour (in ethanol) almost uniformly pale yellow, but cephalic plate, forcipular condyles, tarsungula, and leg pretarsi darker.

*Cephalic capsule*. Cephalic plate subrectangular, ca. 1.4–1.5 times as long as wide, ca. 1.1 times as wide as the forcipular tergite, the lateral margins slightly convex; scutes approximately isometric and up to 10 µm in the anterior half of the cephalic plate, distinctly elongate longitudinally and up to 18 µm long in the posterior half; frontal line absent; setae up to ca. 30 µm long. Clypeus ca. 1.5–1.6 times as wide as long, with lateral margins complete; uniformly areolate, the scutes being up to 10 µm wide, without a distinct clypeal area; a total of 4 setae arranged in two pairs, one anterior to the other. Pleurites uniformly areolate, without setae. Both the intermediate and the lateral parts of the labrum separated from the clypeus by complete sulci; the intermediate part ca. 2.5 times as wide as long, the lateral parts far apart from each other.

*Antennae*. Slender, ca. 3.6 times as long as the head width. Intermediate articles up to ca. 1.2 times as long as wide. Article XIV ca. 2.2 times as long as wide, ca. 1.9–2.0 times as long as article XIII and slightly narrower than the latter. Setae gradually denser and shorter from the basal articles to the distal ones, both ventrally and dorsally, in particular up to 40 µm long on article I but less than 20 µm long on article XIV. Apical sensilla ca. 8 µm long, spear-like, without projections, only gently narrowing at nearly the mid-length. Club-like sensilla ca. 10 µm long, only on article XIV, grouped on the distal parts of both the internal and external sides. Longitudinal rows of 1–3 propioceptive spine-like sensilla at the bases of the antennal articles: two rows on articles I–III, approximately dorsal and ventral; three rows on articles IV–XIV, approximately dorsal, ventro-internal and ventro-external; rows reduced to 0–1 spine on antennal articles VI, X and XIV. A single sensillum, similar to the apical ones, ca. 5 µm long, on both dorso-external and ventro-internal position, close to the distal margin of articles V, IX and XIII.

*First maxillae*. Coxosternite entire, without mid-longitudinal sulcus, without setae. Coxal projection sub-triangular, longer than wide, bearing 2 setae. Telopodite apparently composed of a single article, with 1 seta. Lappets apparently lacking.

*Second maxillae*. Anterior margin of coxosternite concave, without anterior projections. Coxosternite with setae only close to the anterior margin; neither statuminia nor other distinctly sclerotized parts associated to the metameric pores. Telopodite composed of three articles, only slightly narrowing towards the tip, with some distal setae; pretarsus in shape of an elongate claw, more than four times as long as wide at the basis, sub-conic and slightly bent, with a small dorsal bulge.

*Forcipular segment*. Tergite subtrapezoidal, ca. 2.1 times as wide as long, partially covered both by the cephalic plate and the tergite of the first leg-bearing segment, with lateral margins strongly converging anteriorly, posteriorly ca. 0.8 times as wide as the subsequent tergite. Pleurites with sclerotized scapular ridge. Exposed part of the coxosternite ca. as wide as long; anterior margin slightly projecting anteriorly with intermediate part shallowly concave, with a pair of stout, shallow denticles; coxopleural sutures complete, entirely ventral, straight and subparallel for most of their length; chitin-lines apparently absent. Basal distance between the forcipules ca. 0.1–0.2 of the maximum width of the coxosternite. Forcipular trochanteroprefemur ca. 1.6 times as long as wide, the internal side much shorter than the external side, with two mesal denticles, the distal denticle obviously longer than the basal one and slightly bent basalwards. Forcipular intermediate articles distinct, with a mesal shallow bulge each. Tarsungulum ca. 2.8–2.9 times as long as wide, both the external and the internal profiles uniformly curved, but for a mesal moderate basal bulge bearing two denticles, one dorsal to the other; the dorsal denticle longer than all other denticles and distinctly bent basally, not so the ventral denticle; a groove along the internal side of most part of the ungulum, between a dorsal scalloped ridge and a ventral entire ridge. Poison calyx not elongate, in the forcipular intermediate articles.

*Leg-bearing segments*. A total of 33 leg-bearing segments. Metatergite 1 slightly wider than the subsequent one, without pretergite. No paratergites. Metasternites longer than wide, without obvious “carpophagus” pit¸ without glandular pore-fields. Legs of the first pair only slightly smaller than the subsequent ones. Leg claws simple, uniformly bent; a pair of accessory spines, shorter than mid-length of the pretarsus, similar to each other in length.

*Ultimate leg-bearing segment*. Pretergite separated by sulci from pleurites. Metatergite sub-trapezoid, ca. 1.3 times as wide as long, lateral margins convex and distinctly converging posteriorly, posterior margin slightly convex. Metasternite sub-trapezoid, ca. 1.7–1.8 times as wide as long, anteriorly ca. 2.0 times as wide as posteriorly, lateral margins slightly convex and converging backwards; setae distributed uniformly. Coxopleuron ca. 1.7–1.8 times as long of the metasternite; setae distributed uniformly. Coxal organs of each coxopleuron opening through two independent pores, which are approximately aligned along the lateral margin of the metasternite, similar in size, ca. 25–30 µm wide. The telopodite ca. 6–7 times as long as wide, ca. 2.3 times as long and ca. 1.7 times as wide as the penultimate telopodite; six articles, conspicuously swollen, especially prefemur and femur with a disto-ventral bulge each; setae sparse, denser and longer on the ventral distal part of the articles, up to 50 µm long. Pretarsus lacking; a tuft a variously long spines surrounding the tip.

*Postpedal segments*. Genital pleurosternite entire. Gonopods appearing as a pair of basally wide, short laminae. Anal organs relatively large and anal pores exposed.

*Complementary description of mouthparts of another specimen* (PD5768)

*Labrum*. A row of ca. eight very stout tubercles along the posterior margin of the intermediate part. A row of bristles along the posterior margins of the lateral parts.

*Mandibles*. A single pectinate lamella, with ca. 15–20 teeth, on each mandible.

*Intraspecific variability*. Considering a total of nine complete specimens, all females with variously developed gonopodal lamina, the body length varied in the range of 5.7–8.1 mm (measured ± 0.1 mm), the maximum width of the cephalic plate varied in the range of 180–235 µm (measured ± 5 µm) and the number of leg-bearing segments was 33 in four specimens and 35 in five specimens. Some variation was found between specimens also in the elongation of antennae (length/width ratio of the longest intermediate article 1.0–1.2; length/width ratio of article XIV 1.8–2.2) and the forcipules (length/width ratio of trochanteroprefemur 1.5–1.7), the shape of the forcipular denticles (denticles on the trochanteroprefemur more or less pronounced and bent; Fig. 1B), the elongation of the metasternite of the ultimate leg-bearing segment (width/length ratio 1.7–2.0) and the shape of gonopodal lamina (either an entire bilobate lamina or apparently two paired laminae).

### Sundageophilus poriger sp. n.

Taxon classificationAnimaliaGeophilomorphaGeophilidae

http://zoobank.org/690B5097-63CA-44FB-9B8B-6EBFFA2EA9AA

#### Diagnosis.

A *Sundageophilus* species with cephalic plate ca. 1.3 times as long as wide; first maxillary telopodite composed of two articles; no distinct denticles on the trochanteroprefemur; tarsungulum with a single basal denticle; approximately 31 pairs of legs; ventral pore-fields from the first to the penultimate leg-bearing segment; ultimate metasternite ca. 1.5–1.6 times as wide as long, anteriorly ca. 2.6 times as wide as posteriorly, lateral margins almost straight; four coxal pores on each coxopleuron, of which two along the lateral margin of the metasternite; legs of the ultimate pair not swollen in the female, unknown in the male, composed of six articles including distinct trochanter and prefemur, without disto-ventral bulges and without additional dense ventral setae; pretarsus of the ultimate leg pair similar to the other leg claws.

#### Material examined.

*Holotype*. ♀ with gonopods developed, body length 5.8 mm, head width 190 µm; one leg of the ultimate pair damaged; originally entire, subsequently divided into three pieces, (i) head, (ii) forcipular segment, (iii) leg-bearing segments 1–31 and postpedal segments; collected Nov. 2013 by B. Klarner; in ethanol, LIPI macrosoilHJ2c13_chilo165.

*Type locality*. Indonesia, Sumatra, Harapan, jungle rubber agroforest, research site HJ2, 01°49'32"S, 103°17'39"E, ca. 80 m a.s.l., from upper soil layer (0–5 cm).

*Other specimens examined*. 1 ♀, from Bukit Duabelas, jungle rubber agroforest, research site BJ3, 02°03'47"S, 102°48'04"E, ca. 90 m a.s.l., from upper soil layer (0–5 cm), same date and collector as holotype, PD5771; 1 specimen, sex unknown because both anterior and posterior parts missing, from Bukit Duabelas, jungle rubber agroforest, research site BJ5, 02°08'36"S, 102°51'05"E, ca. 50 m a.s.l., from upper soil layer (0–5 cm), same date and collector as holotype, PD5770.

#### Etymology.

“*poriger*” means “bearing pores” and refers to the presence of glandular pore-fields on the ventral side of the trunk.

#### Description.

**Figure 4.**
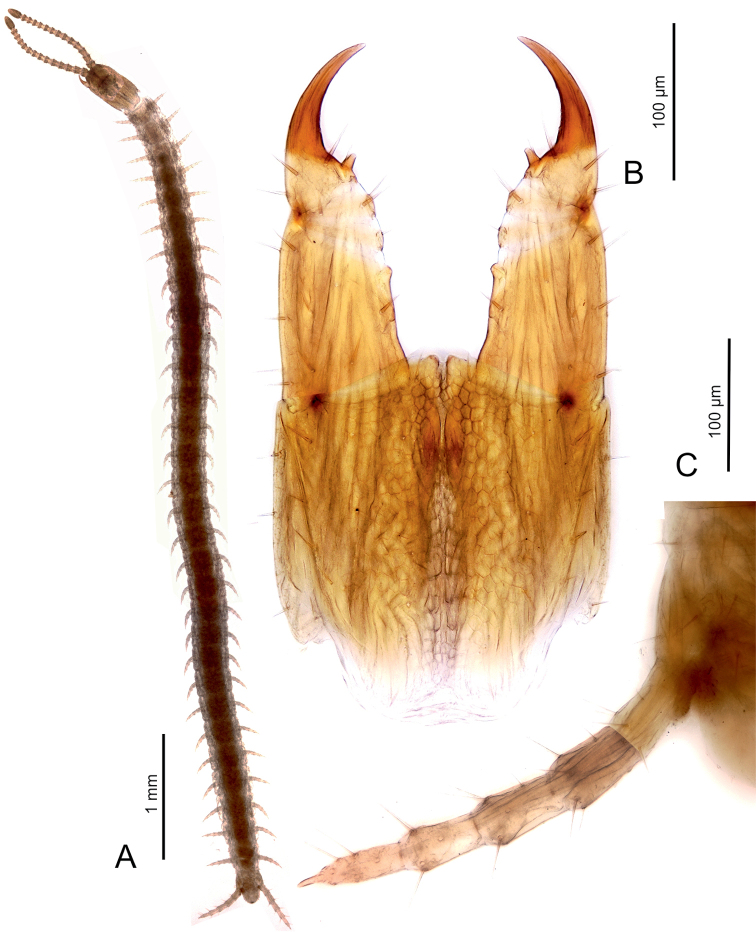
*Sundageophilus poriger* sp. n.: **A** entire body, dorsal view **B** forcipular segment, ventral view **C** ultimate left leg, dorsal view. LM photos of holotype LIPI macrosoilHJ2c13_chilo165.

**Figure 5.**
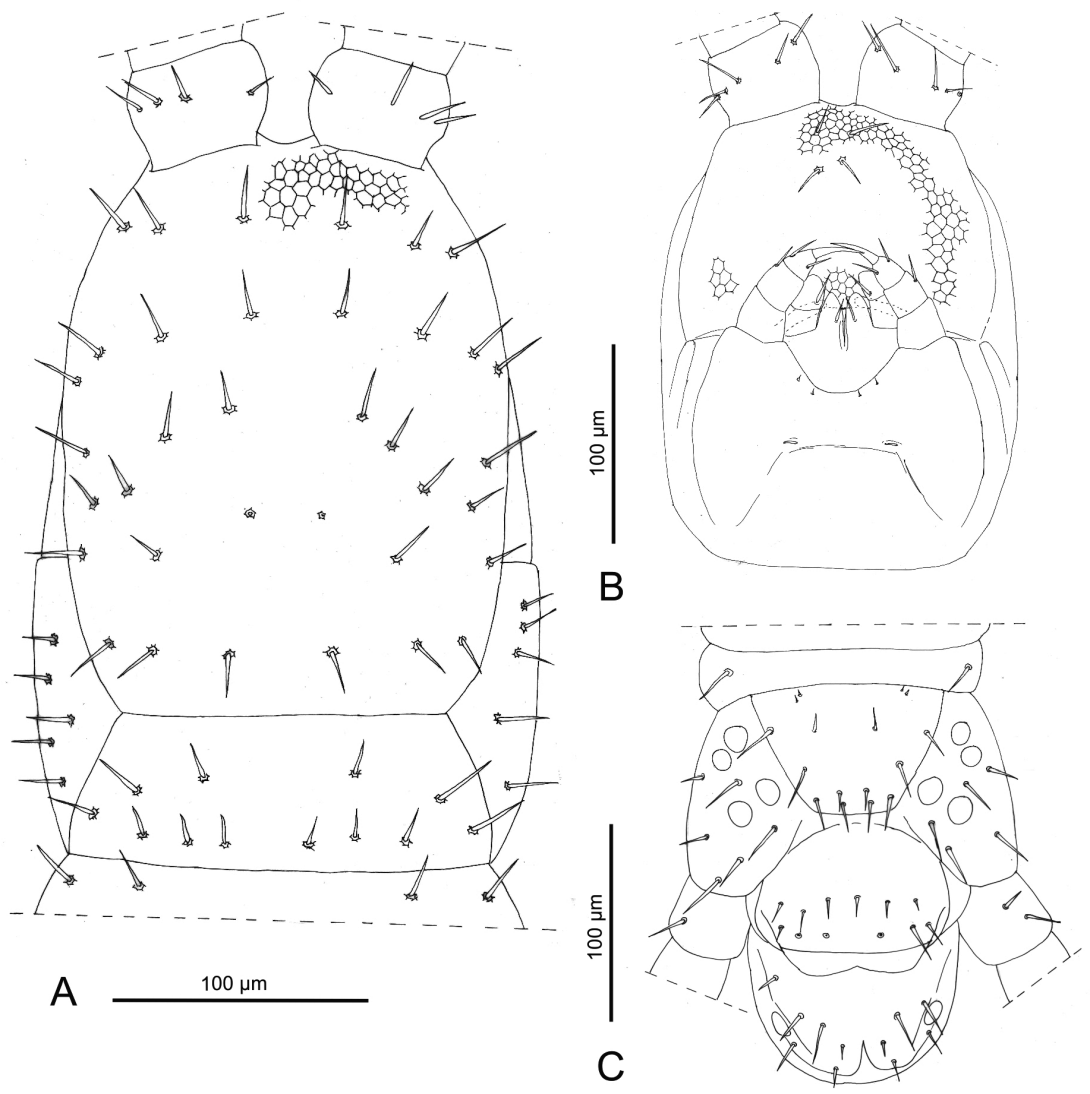
*Sundageophilus poriger* sp. n.: **A** head and forcipular segment, dorsal view, antennal articles II–XIV omitted, areolation only partially drawn **B** head, ventral view, areolation only partially drawn **C** ultimate leg-bearing segment and postpedal segments, ventral view, ultimate legs partially omitted. Line drawings based on LM photos of PD5771 (**A, B**) and holotype LIPI macrosoilHJ2c13_chilo165 (**C**).

*Description of holotype* (♀, LIPI macrosoilHJ2c13_chilo165). See also Figs 4, 5C.

*General features*. Body distinctly narrowing posteriorly. Legs relatively short, most of them ca. 0.2 mm long. Colour (in ethanol) almost uniformly pale yellow, but forcipular tarsungula darker.

*Cephalic capsule*. Cephalic plate subrectangular, ca. 1.3 times as long as wide, ca. as wide as the forcipular tergite, the lateral margins slightly convex; scutes approximately isometric and up to 10 µm in the anterior half of the cephalic plate, indistinct in the posterior half; frontal line absent; setae up to ca. 30 µm long. Clypeus ca. 1.3–1.4 times as wide as long, with lateral margins complete; uniformly areolate, the scutes being up to 10 µm wide, without a distinct clypeal area; two pairs of setae, closed to anterior margin, one anterior to the other. Intermediate part of labrum bearing ca. 8 short tubercles; lateral parts of labrum far apart from each other, separated from the clypeus by complete sulci.

*Antennae*. Slender, ca. 3.7 times as long as the head width. Intermediate articles up to ca. 1.2 times as long as wide. Article XIV ca. 1.9 times as long as wide, ca. 2.1 times as long as article XIII and slightly narrower than the latter. Setae gradually denser and shorter from the basal articles to the distal ones, both ventrally and dorsally, in particular up to 25 µm long on article I but less than 15 µm long on article XIV. Apical sensilla ca. 10 µm long, spear-like, without projections, only gently narrowing at around the mid-length. Club-like sensilla ca. 10 µm long, only on article XIV, grouped on the distal parts of both the internal and external sides. Longitudinal rows of 1–3 proprioceptive spine-like sensilla at the bases of the antennal articles: two rows on articles I–III, approximately dorsal and ventral; three rows on articles IV–XIV, approximately dorsal, ventro-internal and ventro-external; rows reduced to 0–1 spine on antennal articles VI, X and XIV. A single sensillum, similar to the apical ones, ca. 5 µm long, on both dorso-external and ventro-internal position, close to the distal margin of articles V, IX and XIII.

*First maxillae*. Coxosternite without setae. Coxal projection sub-triangular, longer than wide, with a seta. Telopodite composed of two articles, with a seta on the distal one. Lappets lacking.

*Second maxillae*. Anterior margin of coxosternite entire and concave, without anterior projections. Coxosternite with few setae; neither statuminia nor other distinctly sclerotized parts associated with the metameric pores. Telopodite composed of three articles, only slightly narrowing towards the tip, with some distal setae; pretarsus in shape of an elongate claw, ca. 5 times as long as wide at the basis, sub-conic and slightly bent.

*Forcipular segment*. Tergite subtrapezoidal, ca. 1.8 times as wide as long, contiguous to the cephalic plate and partially covered by the tergite of the first leg-bearing segment, with lateral margins strongly converging anteriorly, posteriorly ca. 0.9 times as wide as the subsequent tergite. Pleurites without distinctly sclerotized scapular ridges. Exposed part of the coxosternite ca. 1.1 times as wide as long; anterior margin slightly projecting anteriorly with intermediate part shallowly concave, with short sclerotized denticles; coxopleural sutures straight and subparallel for most of their length; chitin-lines indistinct. Basal distance between the forcipules ca. 0.2 of the maximum width of the coxosternite. Forcipular trochanteroprefemur ca. 1.5–1.6 times as long as wide, the internal side much shorter than the external side, without denticles, only a distal shallow bulge. Forcipular intermediate articles distinct, without denticles. Tarsungulum ca. 2.5–2.6 times as long as wide, both the external and the internal profiles uniformly curved, but for a mesal basal bulge bearing a sub-conic denticle. Poison calyx not elongate, in the forcipular intermediate articles.

*Leg-bearing segments*. 31 leg-bearing segments. Metatergite 1 slightly wider than the subsequent one, without pretergite. No paratergites. Metasternites longer than wide, without obvious “carpophagus” pit, with pore-fields from the first to the penultimate leg-bearing segment. A single, sub-circular, posterior pore-field on each metasternite. Leg claws simple, uniformly bent; at least a posterior accessory spine, much shorter than mid-length of the pretarsus.

*Ultimate leg-bearing segment*. Pleuropretergite without sulci. Metatergite sub-trapezoid, ca. 1.1 times as wide as long, lateral margins convex and distinctly converging posteriorly, posterior margin slightly convex. Metasternite sub-trapezoid, ca. 1.5–1.6 times as wide as long, anteriorly ca. 2.6 times as wide as posteriorly, lateral margins almost straight and converging backwards; setae denser in the posterior part. Coxopleuron ca. 1.7–1.8 times as long as the metasternite; setae distributed uniformly. Coxal organs of each coxopleuron opening through four independent pores, of which two are approximately aligned along the lateral margin of the metasternite, the largest ca. 12 µm wide. The telopodite ca. seven times as long as wide, ca. 1.6 times as long and ca. 1.2 times as wide as the penultimate telopodite; six articles, not swollen; setae sparse, uniformly distributed, up to 50 µm long. Pretarsus claw-like, approximately as long as that of the penultimate legs, apparently without accessory spines.

*Postpedal segments*. Genital pleurosternite entire. Gonopods appearing as a short bilobate lamina. Anal organs relatively large and anal pores exposed.

*Intraspecific variability*. The body length, which is 5.8 mm in the holotype, is estimated to be shorter in the other two specimens, which being damaged cannot be measured accurately. The two specimens with complete trunks are both females with 31 leg-bearing segments.

## Discussion

### Taxonomical remarks

The two new species are confidently recognised as belonging to the family Geophilidae. The combination of a number of characters (pattern of areolation on the clypeus, structure of projections on the labrum, shape of the second maxillary pretarsus and structure of female gonopods) is diagnostic of the Geophilidae in the perception of both traditional taxonomy (e.g., Edgecombe et al. 2011) and recent cladistic analysis (Bonato et al. 2014).

The two species are here described in a new genus because they do not fit the diagnosis of any other known geophilid genus (Table 2) and their morphology does not suggest any obvious relation with other genera. Some characters (elongation of the head, of the second maxillary pretarsus and of the forcipular segment, and number of coxal pores) suggest that *Sundageophilus* may be close to other mainly tropical genera such as *Schizotaenia* Cook, 1896, *Alloschizotaenia* Brölemann, 1909 and *Schizonampa* Chamberlin, 1914, or even to the temperate genus *Plateurytion* Attems, 1909. However, the second maxillae of all species belonging to the latter genera are invariably described and/or illustrated with a medial constriction and distinct sclerotized ridges (so-called statuminia) or rims bordering the metameric pores, also in the smallest species similar in body size to *Sundageophilus*. The elongation of the head, the second maxillary pretarsus, and the forcipular segment is common in two other poorly known genera, namely *Schizonium* Chamberlin, 1955 from South America and *Cephalodolichus* Verhoeff, 1938 from South Africa, but they differ from *Sundageophilus* at least by the denticulate forcipular coxosternite and the densely setose metasternite of the ultimate leg-bearing segment.

**Table 2.** Major differences between the species of *Sundageophilus* gen. n. and all known genera of Geophilidae from south-east Asia and Australasia. Notes: * = number counted on a single or few specimens only.

Genus/Species	General features	Clypeus	Labrum	Second maxillae					Forcipule	Leg-bearing segments		Ultimate pair of legs				
	head and forcipules: distinctly elongate	clypeal area: distinctly present	lateral parts: almost touching each other	coxosternite: statuminia: distinctly present	coxosternite: anterior margin: deeply angulated	coxosternite: anterior projections: distinctly present	pretarsus: much elongate	pretarsus: distinctly stout	tarsungulum: a second denticle flanking the basal denticle	number of leg pairs	anterior metasternites: pore-fields present	coxopleuron: ventral pores opening into pits	coxopleuron: all ventral pores close to metasternite	telopodite: number of articles	telopodite: distinctly swollen in females	pretarsus: shape
*Sundageophilus bidentatus* sp. n.	+	-	-	-	-	-	+	-	+	33–35	-	-	+	6	+	group of spines
*Sundageophilus poriger* sp. n.	+	-	-	-	-	-	+	-	-	31*	+	-	-	6	-	claw
*Geomerinus* Brölemann, 1912	+	+	-	-	+	-	-	-	-	71*	-	-	-	5	-	claw
*Javaenia* Chamberlin, 1944	-	?	-	?	+	-	?	?	-	41–45 *	+	+	?	?	-	claw
*Maoriella* Attems, 1903	+	+	+	-	-	-	+	-	-	41–91	+	+	+	6	-	claw
*Pachymerellus* Chamberlin, 1920	-	-	-	-	+	-	-	-	-	47–65	+	-	+	6	-	claw
*Pachymerinus* Silvestri, 1905	+	+	-	-	+	-	-	+	-	47–81	-	-	-	6	?	claw
*Queenslandophilus* Verhoeff, 1925	+	+	+	+	+	-	-	-	-	37–75	-	-	-	6	-	claw
*Ribautia* Brölemann, 1909	+	+	-	+	+	+	-	-	-	31–125	+	+/-	+/-	6	-	claw/spine
*Sepedonophilus* Attems, 1909	+	+	-	+	+	+	-	+	-	49–79	-	-	-	6	-	claw
*Steneurytion* Attems, 1909	+	+	+	-	+	-	+	-	-	37–53	-	-	-	6	-	claw
*Tuoba* Chamberlin, 1920	-	-	-	-	-	-	-	-	-	39–73	+	+	+	6	-	claw
*Zelanoides* Chamberlin, 1920	+	+	-	?	?	?	?	?	-	33–41	-	-	+	6	-	claw

The two new species are similar to each other in the minute body size, the head and the forcipules distinctly elongate, the second maxillae provided with very slender claws, as well as in other characters. Nevertheless, uniting the two species in a single genus should be taken as a preliminary, parsimonious arrangement. Actually, we cannot rule out the possibility that most similarities between the two species comprise convergent adaptive characters or shared ancestral conditions. As a matter of fact, body miniaturization evolved independently in different lineages of geophilids (Bonato et al. 2015), as happened with the elongation of the head and the forcipules. On the other hand, second maxillae with unusually elongate claws are common in other genera of Geophilidae that are mainly distributed in tropical regions (Table 2), and they evolved independently at least in one species of *Geophilus*, *Geophilus oweni* Bollman, 1887 (Crabill 1954).

### Morphological remarks

The forcipules of *Sundageophilus bidentatus* are unusual in comparison with those of other geophilomorphs: two conspicuous denticles, one dorsal to the other, emerge at the basis of each tarsungulum (Figs 1B, 3).

The forcipules of the geophilomorphs show great diversity in number, size and pattern of denticles (Bonato et al. 2014). The tarsungulum, in particular, is either smooth or provided with a single denticle, which emerges in a sub-basal position on the inner side, sometimes slightly dorsal (Maruzzo and Bonato 2014). In addition to this single basal denticle, other projections are found very rarely; however, in distantly related lineages belonging to all three major clades of geophilomorphs (Bonato et al. 2014): in the mecistocephalid *Takashimaia* Miyosi, 1955, *Anarrup* Chamberlin, 1920 and some species of *Mecistocephalus* Newport, 1843; in the schendyloid *Dinogeophilus* Silvestri, 1909, *Falcaryus* Shinohara, 1970 and some species of *Ityphilus* Cook, 1899; in the geophiloid *Dignathodon* Meinert, 1870 and *Damothus* Chamberlin, 1960. In most of these cases, additional projections emerge distal to the sub-basal denticles, longitudinally aligned along the tarsungulum. Paired sub-basal denticles, one dorsal to the other, are found only in some mecistocephalids (*Anarrup* and some species of *Mecistocephalus*; Bonato and Minelli 2009); however, they are closer to each other and much less conspicuous than those found in *Sundageophilus bidentatus*.

### Biogeographical remarks

The discovery of two geophilid species inhabiting Sumatra is quite unexpected when confronting the known global distribution of the Geophilidae. Up to now, the south-eastern Asia singled out as a major gap in the almost worldwide occurrence of this family, which is the richest and most widespread among the geophilomorph centipedes (e.g., Bonato and Zapparoli 2011).

While many geophilid species in different genera are known living in temperate Asia, Australia and many Pacific islands, only a few claims have been published so far for the entire area comprising Indochina, Malesian islands, and New Guinea, and all these putative records have turned out to refer to misclassified representatives of different families. In particular, the species of *Geoporophilus* Silvestri, 1919 and *Himantosoma* Pocock, 1891 recorded from Sumatra (Table 1) had been originally described as geophilids but they are actually belonging to Gonibregmatidae (Edgecombe et al. 2011). Also, two species of uncertain identity described from Laos (*Luangana varians* Attems, 1953 and *Brachygeophilus robustus* Attems, 1953) had been originally classified between the geophilids, but the described morphological characters are actually inconsistent with Geophilidae, but consistent with Gonibregmatidae. Around Sumatra, the closest undisputable records of Geophilidae are from the Himalayas (*Geophilus intermissus* Silvestri, 1935) and from Bismark and Solomon Islands (*Tuoba sydneyensis* (Pocock, 1891)) (Silvestri 1935, 1936, Jones 1998). However, records of Linotaeniidae, which are morphologically distinct but most probably derived within the Geophilidae (Bonato et al. 2014), are known from northern Laos and Vietnam (species of *Strigamia* Gray, 1843; Attems 1953, Bonato et al. 2012, Maruzzo and Bonato 2014) and apparently also from Java (*Javaenia bataviana* Chamberlin, 1944; Chamberlin 1944; Würmli 1972).

## Supplementary Material

XML Treatment for Sundageophilus

XML Treatment for Sundageophilus bidentatus

XML Treatment for Sundageophilus poriger
